# Continuous Improvement of Chronic Tinnitus Through a 9-Month Smartphone-Based Cognitive Behavioral Therapy: Randomized Controlled Trial

**DOI:** 10.2196/59575

**Published:** 2025-02-18

**Authors:** Uso Walter, Stefan Pennig, Lothar Bleckmann, Kristina Röschmann-Doose, Thomas Wittig, Jörn Thomsen, Winfried Schlee

**Affiliations:** 1 ENT Practice Walter & Zander Duisburg Germany; 2 context Essen-Kettwig Germany; 3 ENT Practice Dr med Bleckmann Kleve Germany; 4 G Pohl-Boskamp GmbH & Co KG Hohenlockstedt Germany; 5 Eastern Switzerland University of Applied Sciences St Gallen Switzerland; 6 Clinic and Polyclinic for Psychiatry and Psychotherapy University of Regensburg Regensburg Germany

**Keywords:** tinnitus, chronic tinnitus, mobile health app, mHealth, mobile app, application, smartphone, digital health, digital technology, digital intervention, cognitive behavioral therapy, randomized controlled trial, statistical analysis

## Abstract

**Background:**

Tinnitus is the perception of sound without an external auditive stimulus and can be a severe burden for affected patients. Medical guidelines recommend cognitive behavioral therapy (CBT) for tinnitus treatment, which effectively improves tinnitus-related distress and anxiety.

**Objective:**

This study investigates the outcome of a 9-month smartphone-based CBT for patients with tinnitus.

**Methods:**

The randomized controlled clinical trial in this study investigates the efficacy of a smartphone-based CBT for 187 patients with chronic tinnitus over a 9-month treatment period. In the initial 3 months, a waiting list design was applied, and in the subsequent study phase, the data of both treatment groups were collectively analyzed. The scores on the Tinnitus Questionnaire (TQ); 9-item Patient Health Questionnaire (PHQ-9); 9-item Self-Efficacy, Optimism, Pessimism (SWOP-K9) questionnaire; and 20-item Perceived Stress Questionnaire (PSQ-20) were assessed as endpoints after 3 and 9 months of treatment.

**Results:**

We observed a statistically significant reduction in the tinnitus burden in patients who received the smartphone-based CBT intervention. Although no changes were observed initially in the TQ sum scores in the waiting control group (baseline mean, 37.8, SD 4.7; 3 months mean 37.5, SD 4.8; analysis of covariance [ANCOVA] *P*=.52), the scores significantly decreased once the app-based CBT had commenced. Data pooled from both groups revealed significant reduction in the TQ sum score by 12.49 (SD 1.44) (ANCOVA, *P*<.001) and 18.48 (SD 1.85) (ANCOVA, *P*<.001) points after 3 and 9 months, respectively, which was also clinically important. The calculated Cohen *d* was 1.38. Similarly, the scores on PSQ-20 (–9.14 points; ANCOVA, *P*<.001), PHQ-9 (–2.47 points; ANCOVA, *P*<.001), and SWOP-K9 (0.17 points; ANCOVA, *P*<.001) were significantly improved at the end of the therapy, with corresponding intermediate effect sizes after 9 months.

**Conclusions:**

The data in our study provide evidence of statistically significant, clinically relevant, and continuous benefits of an app-based CBT intervention in patients with chronic tinnitus.

**Trial Registration:**

Deutsches Register Klinischer Studien DRKS00022973; https://drks.de/search/de/trial/DRKS00022973

## Introduction

Subjective tinnitus is the perception of sound without an external stimulus and is considered chronic if it is present for more than 3 months. The total prevalence of tinnitus among adults is about 14%, which is independent of sex but increases with age and is considered severe in about 2.3% of the affected patients [1]. How the burden is perceived differs substantially depending on the characteristics and severity of tinnitus or accompanying comorbidities and may affect the quality of life substantially. The multifaceted nature of tinnitus, risk factors, and the purely subjective evaluation and accompanying comorbidities make the treatment challenging [2,3]. Highly diverse treatment options have been developed, which may, however, be effective only in a certain subset of patients [4-7]. Treatment may also target not tinnitus itself but comorbidities (eg, hearing loss, depression) with secondary beneficial effects on the patients’ tinnitus [8,9].

In patients with tinnitus resulting from hearing impairment, cochlear implants and hearing aids can significantly improve tinnitus, although variable methodologies applied in clinical trials complicate comparisons between the studies [5,10]. Despite short-term benefits from the intravenous application of lidocaine in some patients, no superiority compared to placebo was observed for commonly prescribed drugs such as betahistine or dexamethasone [8,11]. A study comparing the efficacy of ginkgo extracts and pentoxifylline to reduce tinnitus burden reported comparable decrease in the tinnitus burden for both products. However, reduction in the score of the mini Tinnitus Questionnaire (TQ) was below 10% for both study arms and thus most likely did not reach clinical relevance [12]. Currently, no medicinal products are approved for the treatment of chronic tinnitus. In contrast, pharmacological treatment may be required for comorbidities such as depression or anxiety. A number of therapeutic options have been developed for symptomatic treatment in patients without causative organic dysfunction or unsuccessful treatment. However, clinical evidence for treatments such as transcranial electric or vagus nerve stimulation, hyperbaric oxygen therapy, sound therapy, or tinnitus retraining therapy is scarce, and the evaluation of their efficacy in the clinical guidelines is ambiguous [10].

Among current symptomatic treatment options, cognitive behavioral therapy (CBT) has the highest clinical evidence compared to other treatment approaches. Its validity has been confirmed in several studies, and clinical guidelines recommend CBT for symptomatic treatment [13,14]. Although CBT does not target the tinnitus characteristics (eg, loudness), it focuses on perception and acceptance by the patients and thereby improves their quality of life and comorbidities. In recent years, app- or internet-based telemedicine has become a promising treatment alternative to conventional therapies. The application of CBT, both guided or nonguided by a psychologist or audiologist, is predestinated for remote therapy, and its efficacy to reduce the distress resulting from tinnitus has been proven over the years [15-17]. Nevertheless, more clinical trials are needed to strengthen the clinical evidence, and the research continues [16].

The randomized controlled clinical trial in this study investigates the long-term efficacy of an app-based CBT for tinnitus treatment. The primary endpoint was the difference in the TQ scores between patients who started CBT without delay (group 1) and a waiting list control group (group 2) who started CBT after 3 months, and the results have been previously published [18]. The baseline score of the patients in group 1 using the app improved from 39.7 by 13.4 points but remained largely unchanged (0.6 score points) in group 2. Following the 3 months waiting period, patients of group 2 received the CBT app as well, and patients of both groups continued treatment for a total duration of 9 months, after which final assessments of tinnitus severity (TQ score) as well as stress perception (20-item Perceived Stress Questionnaire [PSQ-20]), depression (9-item Patient Health Questionnaire [PHQ-9]), and self-efficacy (9-item Self-Efficacy, Optimism, Pessimism [SWOP-K9] questionnaire) were performed. This paper presents the long-term results of the full 9-month app-based treatment period in patients with tinnitus of both groups.

## Methods

Our longitudinal analysis represents the outcome of the full 9-month treatment period with a CBT app, marketed under the name Kalmeda. The outcome of the initial 3-month period has been previously published [18].

### Ethics Approval

The clinical investigation plan was prospectively approved by the Ethics Committee of North Rhine, Germany, on May 20, 2020 (case 2020026), following the ISO 14155 norm (Clinical Investigation of Medical Devices For Human Participants: Good Clinical Practice). This study was registered in the German Clinical Trials Register (DRKS-ID DRKS00022973) and conducted in 2 ENT (ear, nose, and throat) centers in North Rhine, Westphalia, Germany.

### Patient Recruitment

Recruitment lasted from August 2020 to March 2021. The follow-up of the last patient ended in March 2022. The eligibility of the patients was assessed during a web-based visit. All patients fulfilled the inclusion criteria age ≥18 years and chronic subjective tinnitus of more than 3 months duration and not the exclusion criterium of an already diagnosed acute or chronic mental illness, whereby tinnitus-related anxiety or depression was no exclusion criterium. All patients signed an informed consent form prior to randomization to one of the treatment arms. The intervention group started using the app immediately after inclusion for a total of 9 months, while the control group commenced treatment after a 3-month waiting period.

### Kalmeda Tinnitus App, Associated Regulations, and Study Design

This study investigates the efficacy of the Kalmeda app in the treatment of chronic tinnitus (Figure S1 in Multimedia Appendix 1). This app follows the principles of CBT, a self-help format following the Zürcher Ressourcen Modell (motivational psychology) and acceptance and commitment therapy for tinnitus [19]. The Kalmeda app was developed by mynoise GmbH and is a CE-marketed medical device since 2019 (DIMDI registration: DE/CA20/mynoise_01/18). During our study, software version 1.5.1 was used. The app was downloaded by users from commonly used app stores and activated by activation codes provided to the patients without a fee. The app has 5 levels with 9 steps each. The first 2 levels of behavioral therapy are typically accomplished within 3 months and the subsequent levels in not less than 7 months.

**Figure 1:**
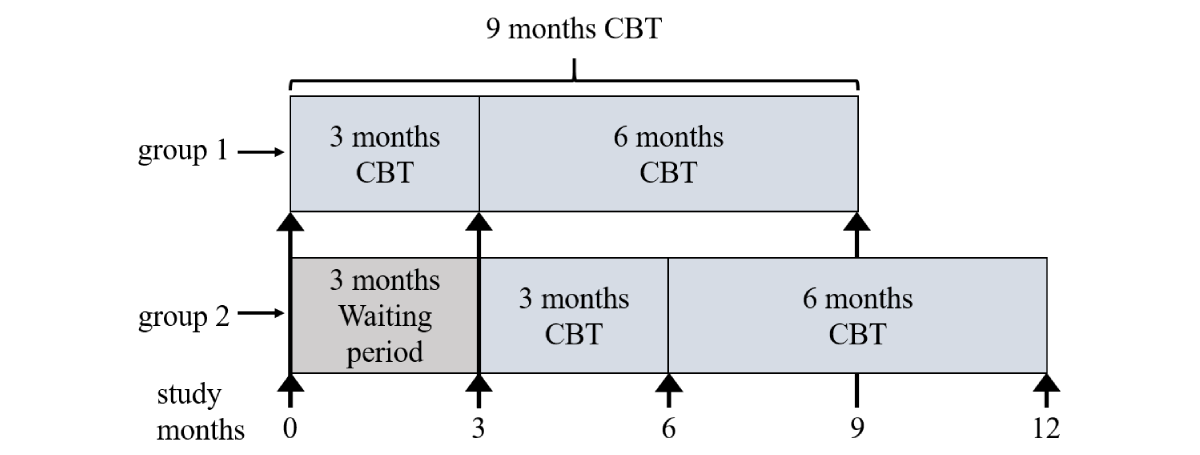
Scheme of the study design. Timepoints of assessments in group 1 and 2 (initial waiting group) are indicated by arrows. CBT: cognitive behavioral therapy.

The efficacy of this intervention was assessed using a randomized controlled clinical trial by applying a waiting group design. The CONSORT-EHEALTH (Consolidated Standards of Reporting Trials of Electronic and Mobile Health Applications and Online Telehealth) checklist was used for reporting of this randomized controlled trial (Multimedia Appendix 2). Patients were randomized to the 2 trial arms in blocks of 6 in the order of their appearance. Although the patients in group 1 started CBT immediately following randomization at T0 (baseline), patients in group 2 postponed treatment and began after the 3-month waiting period (Figure 1). In both groups, the efficacy of CBT was assessed after 3 and 9 months of treatment in comparison to baseline by using paper-based questionnaires sent to the patients by mail. Thus, the total study duration of the patients in group 1 and 2 was 9 and 12 months, respectively. The efficacy endpoints were the changes in tinnitus severity (TQ sum score) [20], depression scores (PHQ-9) [21], stress perception (PSQ-20) [22], and self-efficacy (SWOP-K9) [23] by using validated questionnaires. Published minimal clinically important difference (MCID) values of the TQ score depend on the baseline severity and can vary considerably between 12 for severe baseline values and <5 for lower baseline severity; this study considered the published MCID value of 6.65 [24-27]. The MCID of PHQ-9 varies with severity as well. In mild cases of depression characterized by PHQ-9 scores <10, corresponding to values reported in this study, the MCID is 1-2 points or 14%-22% of the baseline value [28,29]. No MCIDs are published for PSQ-20 and SWOP-K9; thus, generic improvements by 15% of the total scale range were considered as clinical important changes [27]. The severity of tinnitus was assessed according to 4 categories (<30, 31-46, 47-59, and 60-84) from mild to very severe, whereby it is considered as decompensated for TQ scores ≥47 [20]. Once the 3-month waiting period of the control group ended and app-based CBT was initiated, TQ sum scores significantly improved both statistically and clinically in individuals of the control group to a similar extent as in the intervention group. As data from baseline and after 3 and 9 months of treatment did not differ significantly between the groups, the data for all the patients using the app were pooled for our further analysis and evaluated collectively, independent from the initial randomization into group 1 or 2.

### Statistical Analysis

Sample size calculations were based on an effect size of 0.5 and a power of 0.8 by using a 2-sided *t* test with an α level of .05. The assumed intermediate effect size is based on the range of efficacy of CBT reported in tinnitus studies [30-33]. This agreed to a sample size of 64 per group or 128 patients in total. Considering a dropout rate of 15%, a total of at least 150 patients needed to be enrolled. All statistical analyses were performed with SAS 9.4 (SAS Institute Inc).

The primary statistical analysis was performed by applying analysis of covariance (ANCOVA). Missing data were imputed using reference-based multiple imputations. Robustness was verified with a sensitivity analysis by using a completer data set, which encompassed only individuals without any missing answers in particular questionnaires over the whole period. Thus, the numbers of completers vary between the different endpoints (TQ: n=88, PHQ-9: n=86, PSQ-20: n=86, and SWOP-K9: n=91). The outcomes were analyzed in comparison to baseline by using the paired 2-sided *t* test. Furthermore, Pearson correlations were performed for changes of TQ and the 3 secondary scores. The distribution of the demographic and clinical characteristics of both groups at baseline were tested for normality by using Shapiro-Wilk test and for variance homogeneity by using Levene test. Depending on the outcome of the tests, group comparisons were performed using nonparametric (Mann-Whitney *U* test, chi-square) or parametric analyses (*t* test). Frequency changes in tinnitus severity categories were analyzed using chi-square tests. Values in the text are represented as mean (SD).

## Results

### Patient Demographics and Disposition

**Figure 2:**
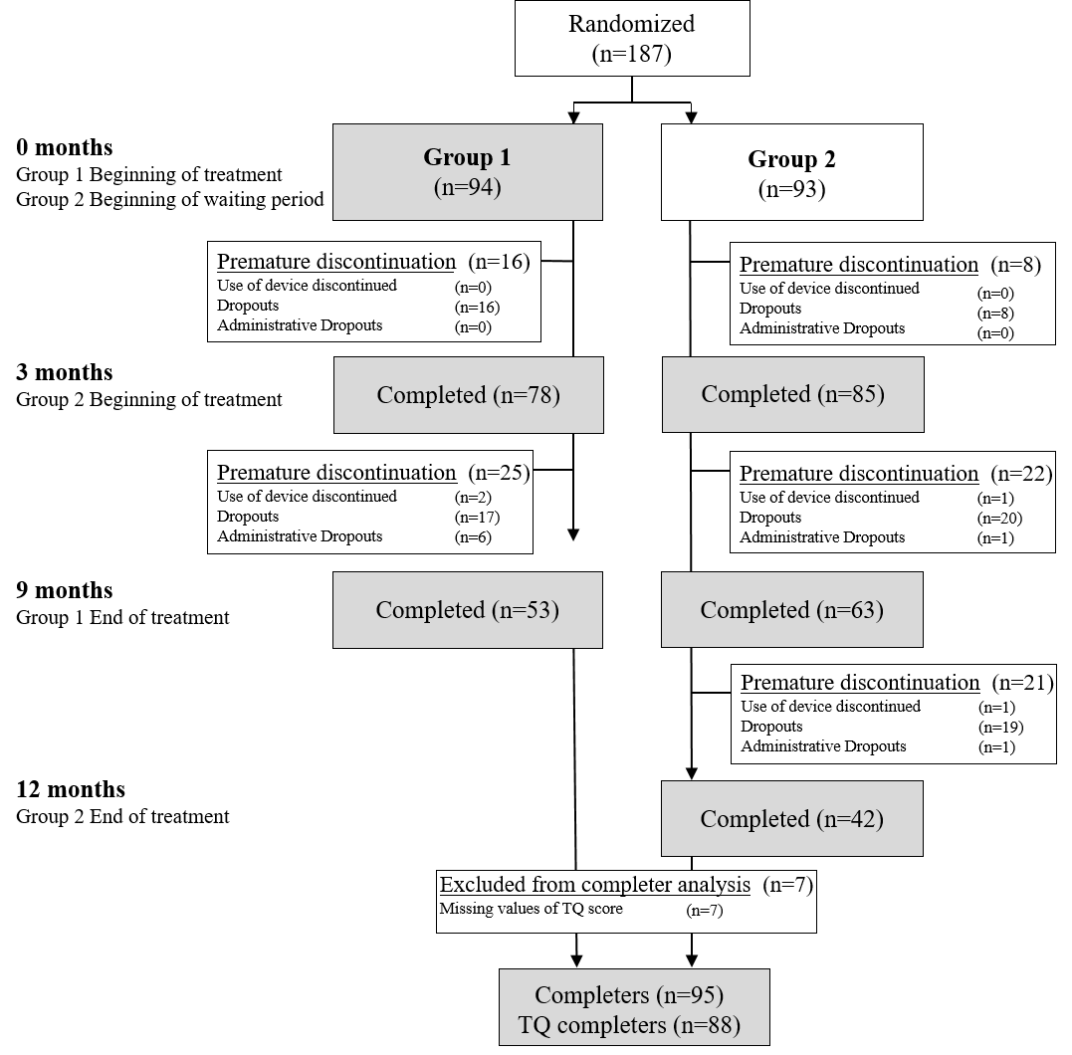
CONSORT (Consolidated Standards of Reporting Trials) diagram. Disposition of the patients in this trial. The term “completers” encompasses all patients with complete entries without any missing data in the Tinnitus Questionnaire. TQ: Tinnitus Questionnaire.

A total of 187 patients were randomized in blocks of 6 to group 1 or 2 (Table 1). Group 1 started using the app immediately after randomization, whereas patients in group 2 were assigned to a 3-month waiting period before treatment commenced. In group 1, 53 patients completed the treatment. In group 2, 42 patients completed the treatment. Thus, a total of 95 patients completed the study after 9 treatment months (Figure 2). At baseline, groups 1 and 2 did not differ with regard to mean age (48.2 years, SD 12.5 years), sex ratio (males, 97/187, 51.9%; females, 90/187, 48.1%), and duration of tinnitus (mean 6.57 years, SD 6.93 years). The mean duration of the app-based therapy was comparable in both study arms and was 99.3 (SD 14.4) and 288.1 (SD 29.0) days for the 3- and 9-month treatment period, respectively. Tinnitus distress was measured with TQ scores as the primary outcome. Secondary outcomes were measured with PSQ-20, PHQ-9, and SWOP-K9 questionnaires (Table 1).

**Table 1:** Demographic and clinical characteristics of the participants at baseline (N=187).

			Group 1 (n=94)		Group 2 (n=93)		*P* value	
**Participants**								.27^a^
	Males, n (%)	45 (48)		52 (56)				
	Females, n (%)	49 (52)		41 (44)				
Age (years), mean (SD; range)			48.1 (12.8; 22-72)		48.4 (12.2; 21-74)		.86^a^	
Tinnitus duration (years), mean (SD; range)			6.21 (6.62; 0.33-28)		6.94 (7.25; 0.25-31)		.79^b^	
Tinnitus Questionnaire sum score, mean (SD; range)			39.65 (15.08; 12-73)		38.30 (15.10; 9-76)		.54^a^	
Perceived Stress Questionnaire-20 items, mean (SD; range)			46.9 (17.6; 11.7-95)		45.1 (18.9; 11.7-83.3)		.51^a^	
Patient Health Questionnaire-9 items, mean (SD; range)			8.31 (3.89; 2-19)		7.53 (4.21; 0-21)		.19^a^	
Self-Efficacy, Optimism, Pessimism questionnaire-9 items, mean (SD; range)			2.76 (0.45; 1.8-4)		2.75 (0.52; 1.4-3.8)		.92^a^	

^a^Paired 2-sided *t* test.

^b^*U* test.

### Primary Outcome

**Figure 3:**
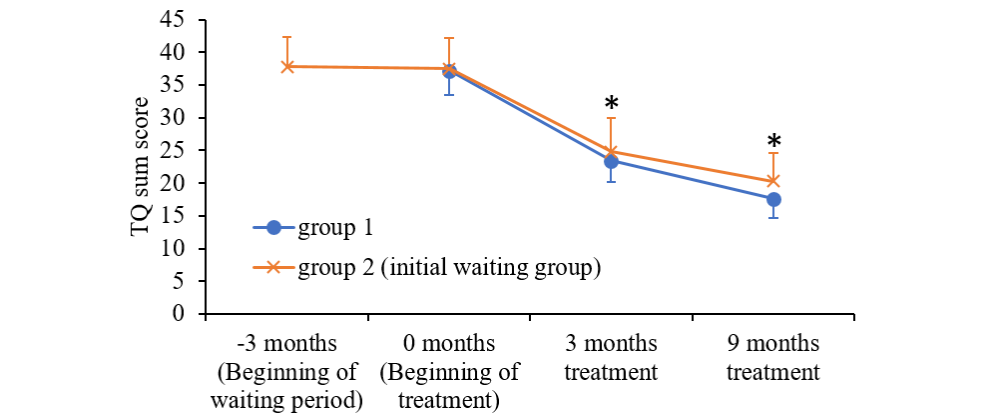
Tinnitus questionnaire sum score of completers in group 1 (blue) and 2 (orange) during the waiting (group 2 only) and the app-based treatment period. For improved clarity of the treatment effect, the improvement during the app-based treatment period is shown in parallel for both groups. Significant improvements compared to baseline are indicated by stars. TQ: Tinnitus Questionnaire.

The app-based CBT significantly improved the subjective tinnitus burden, as assessed by the TQ sum scores in patients of both study arms after 3 and 9 months of treatment, whereas the TQ scores of patients of group 2 did not improve during the waiting period (Table 2, Figure 3). During treatment, the TQ sum scores of the completers declined significantly from 37.27 (SD 13.49) at baseline to 23.99 (SD 13.50) (paired t_87_=10.75; *P*<.001) and 18.73 (SD 11.48) (paired t_87_=13.0; *P*<.001) after 3 and 9 months of treatment, respectively.

**Table 2:** Tinnitus Questionnaire sum score of the completers at the beginning of the waiting period (group 2 only), beginning of treatment, and after 3 and 9 months of treatment.

Timepoint of the assessments	Group 1 (n=51), mean (SD)	Group 2 (n=37), mean (SD)	*P* value
Beginning of the waiting period	N/A^a^	37.8 (14.0)	N/A
Beginning of the treatment	37.1 (13.0)	37.5 (14.3)	.54^b^
3 months	23.4 (11.8)	24.8 (15.7)	<.001
9 months	17.6 (10.2)	20.3 (13.0)	<.001

^a^N/A: not applicable.

^b^Paired 2-sided *t* test.

In agreement, the imputation of missing data by using multiple imputations resulted in initial TQ sum scores of 38.46 (SD 15.49), which improved to 25.97 (SD 14.03) after 3 months and to 19.98 (SD 9.32) after 9 months of CBT in the analysis of the completed courses corresponding to a total improvement of 12.49 (SD 9.65) after 3 months (*P*<.001) and 18.48 (SD 12.43) after 9 months (*P*<.001) during therapy for all the patients (Table 3). Thus, the prolonged treatment period of another 6 months resulted in a further and significant decrease of TQ scores.

**Table 3:** Analysis of covariance of treatment difference compared to baseline of Tinnitus Questionnaire sum scores after multiple imputations at 3 and 9 months after the beginning of the treatment.

Parameter	Estimate	SE	*P* value	95% confidence limits	
				Lower limit	Upper limit
Intercept	–0.99	2.00	.62	–4.94	2.94
∆M3^a^ (M3 – baseline)	–12.49	0.84	<.001	–14.14	–10.84
∆M9^b^ (M9 – baseline)	–18.48	1.08	<.001	–20.63	–16.33
Baseline	–0.45	0.05	<.001	–0.55	–0.35
∆M9 – ∆M3	–5.99	1.16	<.001	–8.28	–3.70

^a^Score difference after 3 months of treatment compared to baseline.

^b^Score difference after 9 months of treatment compared to baseline.

The calculated effect size Cohen *d* was 1.15 and 1.38 after 3 and 9 months of therapy, respectively, corresponding to a strong effect, whereby improvement correlated with severity at baseline (*r*=–0.48) and after the first 3 months (*r*=–0.53) being much more pronounced in patients severely affected by tinnitus. The improvement of the mean TQ sum score was accompanied by significant reductions in the incidence of decompensated tinnitus (TQ sum score≥47). Among completers, the number of patients with decompensated tinnitus was reduced from the initial 22 to 7 after 3 months (*χ*^2^_1_=9.3; *P*=.002) and to only 1 patient after 9 months of treatment (*χ*^2^_1_=22.1; *P*<.001). Not very severe cases were remaining, but patients had improved to lower severity categories. In agreement, the number of patients characterized by mild tinnitus severity (TQ≤30) increased from 25 at baseline to 63 and 73 after 3 and 9 months, respectively (*χ*^2^_1_=32.8 and 53.1, respectively; *P*<.001; Figure S2 in Multimedia Appendix 1). After 3 months, 68% (60/88) responded to the treatment, and after 9 months of therapy, 80% (70/88) responded to the treatment, as their TQ sum score improved by more than the MCID value of 6.65. The baseline TQ score and its improvement during the first 3 months of therapy and its impact on completion or premature discontinuation of therapy was assessed. An initial worsening of symptoms does not seem to result in premature termination, as this was more often observed in completers (7 patients) than in dropouts (3 patients).

Individuals with mild and intermediate tinnitus (<46 points) at baseline documented average improvements by about 10 points (or 35% of baseline severity) within the first 3 months of treatment and thereby reached TQ scores of about 20 (Figure S3 in Multimedia Appendix 1). Approximately equal numbers of patients either terminated participation prematurely (55 patients) or completed treatment (66 patients). In contrast, patients with severe tinnitus (≥47) either experienced strong improvements of 18 points in the TQ score (35% of baseline severity) and completed treatment (22 patients) or small improvements of 10 points in the TQ score (equivalent to 18% improvement when compared to baseline severity), and treatment was terminated prematurely (13 patients).

### Secondary Outcomes

Aside from the TQ sum score, the secondary outcomes, that is, stress, depression, and self-efficacy were assessed. The beneficial effects of our app-based CBT were observed in PSQ-20 scores over the whole treatment period. Significant reductions became evident already after 3 months (–4.62 points), and the score improved even further by –9.14 after 9 months of treatment (ANCOVA, *P*<.001). Similar significant changes (–5.66 and –9.55) were present in the completer data after 3 and 9 months as well (paired *t* test, *P*<.001), respectively, without reaching the clinically important difference. The effect sizes were 0.45 after 3 months and 0.60 after 9 months of CBT. Thus, the initially small effect became intermediate in the second treatment phase. Individual changes in PSQ-20 scores were well-correlated with TQ improvement after 3 (*r*=0.57) and 9 (*r*=0.58) months, respectively (Tables S1 and S2 in Multimedia Appendix 1).

The PHQ-9 score, as a measure of depression severity, decreased significantly by 1.40 and 2.47 points (ANCOVA, *P*<.001) after 3 and 9 months, respectively, as evaluated using multiple imputation. The sensitivity analysis using the completer data set yielded comparable significant results starting with 7.77 (SD 4.00) at baseline and lower values of 6.29 (SD 3.48) and 5.42 (SD 3.07) following treatment (paired *t* test, *P*<.001). Based on the published MCID, PHQ-9 changes were considered clinically relevant. The corresponding effect sizes were 0.52 and 0.68 after 3 months and 9 months, respectively, representing intermediate effects (Tables S1 and S2 in Multimedia Appendix 1). Changes in TQ and PHQ-9 were correlated after 3 (*r*=0.57) and 9 (*r*=0.58) months of therapy.

Self-efficacy was assessed using SWOP-K9. Imputed data revealed no significant improvement after 3 months (0.04, ANCOVA, *P*=.16) but after 9 months of treatment (0.17, ANCOVA, *P*<.001). Completers were characterized by mean baseline values of 2.77 (SD 0.51). Although no significant improvement was evident after 3 months (2.78, SD 0.54; paired *t* test *P*=.75), statistically significantly higher values of 2.95 (SD 0.47) were obtained after 9 months (paired *t* test, *P*<.001); however, the improvement did not reach the generic MCID of 15%. The 9-month effect size was 0.5 corresponding to an intermediate effect (Tables S1 and S2 in Multimedia Appendix 1). Only low correlations between individual changes of TQ score and SWOP-K9 were obtained after 3 months (*r*=–0.22), which increased after 9 months (*r*=0.54). No adverse events were reported during this clinical study.

## Discussion

### Principal Results

This study reports the outcome of a 9-month treatment with an app-based CBT for chronic tinnitus. This study is a follow-up to the previously published data of the initial 3-month treatment period [18]. The 9-month data confirm highly beneficial improvements in the tinnitus burden by CBT with the Kalmeda app. Within 3 months of treatment, the TQ sum scores improved significantly. There are currently 3 studies on the MCID of the TQ scores available. Adamchic and colleagues [24] reported an MCID of 6.65 (SD 7.99) points (mean follow-up time, 44 days) [24]. Hall and colleagues [25] reported an MCID of 12 (SD 9.8) points (follow-up time, 5 days) [25] and 7.5 points for the Mandarin version (follow up time, 6 months) [26]. After 3 months of web-based CBT, the average improvement in the tinnitus burden surpassed the average thresholds, and during the long-term treatment, tinnitus symptoms continued to improve even further, leading to an additional decrease by 5.99 points in the TQ score. Importantly, as perceived improvement of tinnitus is independent of the duration of treatment but depends on the baseline value, it is smaller in patients with lower initial burden [24]. The MCID for mild tinnitus (TQ≤30) corresponds to 3.17 (SD 7.15); this is much lower than the mean value of both MCID criteria [24,25]. After the first 3-month treatment period, patients were characterized by mean TQ scores of 23.99; thus, it is likely that further amelioration of their tinnitus symptoms experienced during the complete 9-month treatment is considered clinically relevant. Our study data reveal that the efficacy of the app-based CBT in the reduction of the tinnitus burden is comparable to that of treatments such as neuromodulation or transcranial direct current stimulation [6,34].

The comparison of active treatment to a waiting list control group is a common study design; yet, the assessment of the long-term outcome is difficult once the control group receives the same treatment. In this trial, the efficacy of CBT was compared to the waiting list control group, which remained largely unchanged during the initial 3-month period, but no control group was present during the full 9-month treatment period [18].

In order to compensate for the lack of an internal control, treatment efficacy in this study was compared to the outcomes of untreated control groups in randomized clinical trials with comparable durations. Few studies [35-39] have reported TQ sum scores in patients not receiving adequate treatment over several months. In a randomized trial testing the efficacy of tinnitus retraining therapy, no improvement was observed for patients in the waiting list control group after 12 months [35]. No improvement but a tendency of worsened symptoms was observed in patients in the waiting list control over a period of 6 months [36]. In patients receiving no treatment, the Tinnitus Severity Index was largely unchanged after 6 and 12 months, corresponding to an effect size of 0.0 and 0.1 [37]. In patients in the waiting list group during a 24-week trial, improvement in the TQ sum score corresponded to an estimated effect size below 0.4 [38]. Evidence from literature suggests only minor changes in tinnitus severity in untreated controls and a maximum effect size up to 0.4 corresponding to at most a small effect according to the definition by Cohen [39]. In comparison, the effect size of the app-based CBT group was 1.15 after 3 months and increased even further to 1.38 after 9 months of therapy. Thus, long-term app-based CBT in this trial resulted in pronounced improvement, which exceeded the expected outcome in the absence of any treatment in studies of comparable length by far. In addition, a waiting control group design does not allow to evaluate a potential placebo effect. In trials investigating medicinal products or medical devices, the efficacy of placebo treatment can be of relevance but at the same time is smaller by a factor of 3 than the improvement observed in the recent studies [40,41].

Dropouts can be a problem for the outcome of clinical trials. In this trial, 12.8% (24/187) of the patients stopped participation during the initial 3-month period until the primary endpoint was reached. In the following study period from 3 to 9 months, the dropout rates increased, resulting in a total loss of follow-up of 52.9% (99/187). A comparable rate of 51% was reported in a previous study applying internet-based CBT for tinnitus [15]. In general, dropout rates in studies using app-based treatments (~43%) often exceed rates common for studies applying in-person treatment [42]. However, despite the risk of therapy attrition, treatment apps are advantageous, for example, they are readily available without extended waiting periods, can be used 24/7, and are available even in remote areas.

Improvement of tinnitus-related distress within the first 3 months of app-based CBT did not differ significantly between dropouts and completers, suggesting that the patient’s decision to prematurely end treatment was based on either treatment success (an individual had either reached an acceptable level of tinnitus severity or experienced considerable relief of its burden) or treatment failure (as defined by unsatisfying improvement or the burden for the treatment was too high). As in most cases, no further information on the reasons for dropouts were given; therefore, we cannot be sure about the reason with absolute certainty, but an analysis of the data after 3 months of treatment supports this hypothesis. Interestingly, in patients with mild and intermediate tinnitus (<46 points) who reached an average TQ score of about 20 within the first 3 months, subsequently, it was merely a 50/50 chance if they dropped out or completed treatment. Patients may have continued therapy even longer and thus reach even lower scores before prematurely dropping out; unfortunately, detailed information is not available. In contrast, patients with more severe burden at baseline did not reach such low TQ levels within 3 months. However, the majority of them experienced a pronounced and clinically relevant reduction of 18.9 points, which seemed to motivate continuation of therapy. Indeed, these patients finally reached TQ scores of 25.8 points after 9 months. A smaller number of 13 patients had improved to a lesser extent by 10 points (18% of baseline), thus still reaching the MCID and discontinued later. For the latter, it seems likely that unsatisfactory treatment was the major reason for the dropout. Thus, the data suggest that premature termination can result from subjective evaluation of success or failure of therapy depending on the relative improvement. The same may apply during the second study phase; however, the pronounced increase in dropouts suggests that the long study duration might have contributed to premature terminations as well. This is supported by the higher dropout rate of patients from the waiting list control group (51/93, 55%) whose involvement in the trial lasted for a full year when compared with the intervention group (41/94, 44%) who finished treatment 3 months earlier.

Patients with tinnitus are often characterized by elevated depression and stress perception, which was measured with PHQ-9 as well as PSQ-20, respectively [43,44]. Following the 3-month treatment, PHQ-9 and PSQ-20 measures had significantly improved. Both parameters decreased even further during the prolonged treatment, and improvements became clinically relevant. As suggested by Wallhauser-Franke and colleagues [45], the measures of tinnitus loudness and tinnitus distress need to be distinguished. The app-based CBT in our study did not improve perception of tinnitus loudness but significantly reduced tinnitus distress. Improvement of PHQ-9 and PSQ-20 scores after CBT therefore are in parallel to the observed reduction of the TQ sum score observed after 3 and 9 months of treatment.

In contrast to the 2 previous secondary endpoints, self-efficacy assessed by SWOP-K9 was almost unchanged following 3 months of CBT, but a statistically significant improvement was evident after 9 months, albeit clinically relevant levels were not reached. This highlights the importance of prolonged treatment beyond the initial 3 months period, leading to significant improvements of tinnitus burden on all tested scales.

### Limitations

This study has limitations. The waiting list design allows an accurate quantification of treatment effects of CBT in comparison with the untreated control but lacks an internal control for the whole 9-month treatment period. Thus, the superiority of the treatment had to be verified by comparison to studies in this indication with comparable duration. Furthermore, a potential placebo effect was not assessed and therefore cannot be ruled out, but is likely to be relatively small [40,41]. The tinnitus distress of the included study participants was assessed with an average TQ sum score of 39.6 in group 1 and 38.3 in group 2. Patients with this amount of tinnitus distress are considered as grade 2 patients (out of 4 grades). This study shows that patients with chronic tinnitus of this grade of severity can significantly benefit from the app-based treatment. At the current state of research, the results cannot be generalized to patients with very severe amount of tinnitus distress (grade 4).

### Conclusions

This study reveals the pronounced effect of an app-based CBT on tinnitus distress, and in the course of treatment, the number of patients affected by decompensated tinnitus decreased significantly. In addition, the majority were characterized by mild tinnitus severity during the final assessment.

## Appendix

Multimedia Appendix 1 Supplementary data.

Multimedia Appendix 2 CONSORT-EHEALTH checklist (V 1.6.1).

## Abbreviations

ANCOVA: analysis of covariance
CBT: cognitive behavioral therapy
CONSORT-EHEALTH: Consolidated Standards of Reporting Trials of Electronic and Mobile Health Applications and Online Telehealth
ENT: ear, nose, and throat
MCID: minimal clinically important difference
PHQ-9: 9-item Patient Health Questionnaire
PSQ-20: 20-item Perceived Stress Questionnaire
SWOP-K9: 9-item Self-Efficacy, Optimism, Pessimism questionnaire
TQ: Tinnitus Questionnaire
