# Supplementary material for: Continuous Improvement of Chronic Tinnitus Through a 9-Month Smartphone-Based Cognitive Behavioral Therapy: Randomized Controlled Trial
Source: J Med Internet Res. 2025 Feb 18;27:e59575. doi: 10.2196/59575 (PMC11888023; doi:10.2196/59575)
Supplement: Multimedia Appendix 1 [file jmir_v27i1e59575_app1.docx]

**Supplementary information**


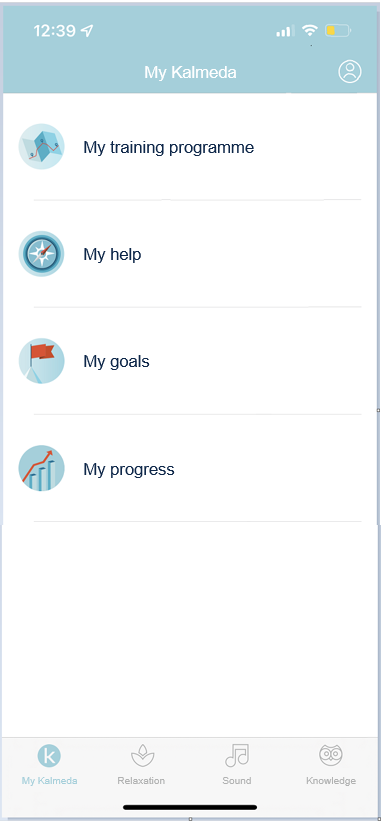


Supplementary Figure S1: Screenshot of the home screen of Kalmeda


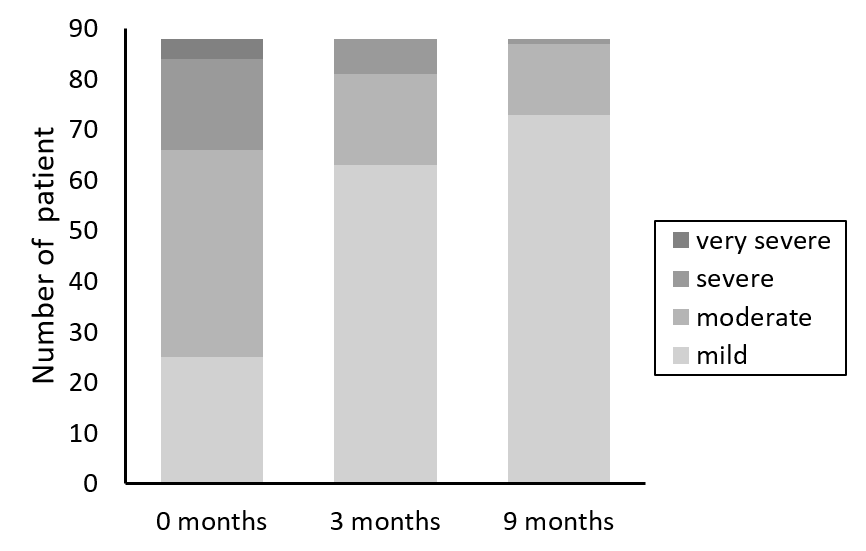


Supplementary Figure S2: Number of patients (completers) per severity category (mild <30, moderate 31-46, severe 47-59 and very severe 60-84) before and after 3 and 9 months of treatments Reduction of TQ sum score of completers after 3- and 9-months of treatment categorized based on patients’ initial severity categories. Mean ± SD


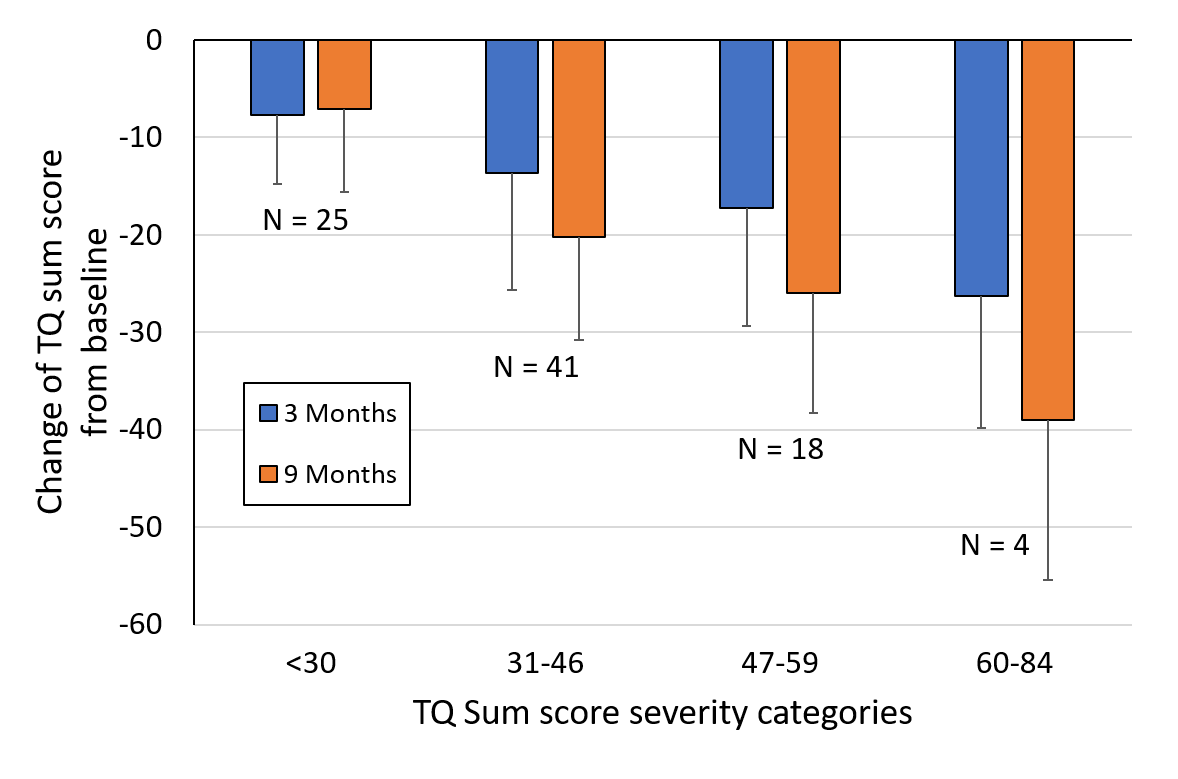


Supplementary Figure S3: Reduction of TQ sum score of completers after 3- and 9-months of treatment categorized based on patients’ initial severity categories. Mean ± SD

Supplement Table S1: ANCOVA of treatment differences for PSQ20 sum score, PHQ9 sum score and SWOP K9 compared to baseline after multiple imputation 3- and 9-months after begin of treatment:

1. PSQ20 sum score

| Parameter | Estimate | SE | *P* value | 95% CI | |
| --- | --- | --- | --- | --- | --- |
|  |  |  |  | Lower limit | Upper limit |
| Intercept | 3.28 | 2.50 | .1903 | -1.64 | 8.19 |
| ∆M3^a^ (M3 – baseline)  ∆M9^b^ (M9 – baseline)  Baseline | -4.62  -9.14  -0.27 | 0.99  1.39  0.05 | <.001  <.001  <.001 | -6.55  -11.88  -0.36 | -2.68  -6.40  -0.18 |
| ∆M9 - ∆M3 | -4.52 | 1.47 | .0026 | -7.43 | -1.61 |

1. PHQ9 sum score

| Parameter | Estimate | SE | *P* value | 95% CI | |
| --- | --- | --- | --- | --- | --- |
|  |  |  |  | Lower limit | Upper limit |
| Intercept | 1.06 | 0.42 | .0123 | 0.23 | 1.89 |
| ∆M3^a^ (M3 – baseline)  ∆M9^b^ (M9 – baseline)  Baseline | -1.40  -2.47  -0.44 | 0.21  0.27  0.04 | <.001  <.001  <.001 | -1.81  -3.00  -0.52 | -1.00  -1.94  -0.35 |
| ∆M9 - ∆M3 | -1.07 | 0.29 | <.001 | -1.65 | -0.48 |

1. SWOP K9

| Parameter | Estimate | SE | *P* value | 95% CI | |
| --- | --- | --- | --- | --- | --- |
|  |  |  |  | Lower limit | Upper limit |
| Intercept | 0.89 | 0.12 | <.001 | 0.66 | 1.13 |
| ∆M3^a^ (M3 – baseline)  ∆M9^b^ (M9 – baseline)  Baseline | 0.04  0.17  -0.26 | 0.03  0.04  0.04 | .1647  <.001  <.001 | -0.02  0.10  -0.34 | 0.10  0.24  -0.185 |
| ∆M9 - ∆M3 | 0.13 | 0.04 | .0063 | 0.04 | 0.21 |

^a^score difference after 3 months of treatment compared to baseline

^b^score difference after 9 months of treatment compared to baseline

Supplement Table S2: Secondary study parameters at baseline and after 3 and 9 months of treatment in patients with complete data documentation of the respective questionnaire (completers):

1. PSQ20 sum score (Completer) N = 86

|  | baseline | 3 months | 9 months |
| --- | --- | --- | --- |
| Mean (95% CI) | 45.33 (4.32) | 39.67 (4.12) | 35.78 (3.98) |
| Results Paired t-test vs. baseline |  | t = 4.14, P <.001 | t = 5.53, P <.001 |
| Effect size |  | 0.45 | 0.60 |

1. PHQ9 sum score (Completer) N = 86

|  | baseline | 3 months | 9 months |
| --- | --- | --- | --- |
| Mean (95% CI) | 7.77 (0.86) | 6.29 (0.75) | 5.42 (0.66) |
| Results Paired t-test vs. baseline |  | t = 4.81, P <.001 | t = 6.26, P <.001 |
| Effect size |  | 0.52 | 0.68 |

1. SWOP-K9 (Completer) N = 91

|  | baseline | 3 months | 9 months |
| --- | --- | --- | --- |
| Mean (95% CI) | 2.77 (0.11) | 2.78 (0.11) | 2.95 (0.10) |
| Results Paired t-test vs. baseline |  | t = -0.31 P =.75 | t = -4.80 P <.001 |
| Effect size |  | --- | 0.50 |
